# Clinical Practice Experiences in Diagnosis and Treatment of Traumatic Brain Injury in Children: A Survey among Clinicians at 9 Large Hospitals in China

**DOI:** 10.1371/journal.pone.0142983

**Published:** 2015-11-13

**Authors:** Fei Di, Qi Gao, Joe Xiang, Di Zhang, Xiuquan Shi, Xueqiang Yan, Huiping Zhu

**Affiliations:** 1 Department of Neurosurgery, Beijing Tian Tan Hospital, Capital Medical University, Beijing, China; 2 Department of Epidemiology and Health Statistics, School of Public Health, Capital Medical University, Beijing, China; 3 Center for Injury Research and Policy, The Research Institute at Nationwide Children’s Hospital, The Ohio State University College of Medicine, 700 Children’s Drive, Columbus, Ohio, United States of America; 4 Department of Neurosurgery, Beijing Children’s Hospital, Capital Medical University, Beijing, China; 5 Department of Epidemiology and Health Statistics, School of Public Health, Zunyi Medical University, Zunyi, Guizhou, China; 6 Department of Neurosurgery, Wuhan Children’s Hospital, Wuhan, Hubei, China; Institute of Automation, Chinese Academy of Sciences, CHINA

## Abstract

Proper diagnosis and treatment of traumatic brain injury (TBI) in children is becoming an increasingly problematic issue in China. This study investigated Chinese clinicians to provide information about their knowledge and experiences in diagnosis and treatment of pediatric TBI. We conducted a questionnaire survey among clinicians in the emergency departments and neurosurgery departments at 9 major hospitals in China. The questionnaire included demographic information, and knowledge and experiences regarding the diagnosis and treatment of pediatric TBI. A total of 235 clinicians completed questionnaires. 43.8% of the surveyed clinicians reported children with only scalp hematoma without any other signs and symptoms of concussion as TBI cases. Most clinicians (85.1%) reported no existing uniform diagnostic criteria for children with TBI in China. The majority of clinicians (91.9%) reported that CT scans were performed in all patients with suspected head injury as a routine procedure in their hospitals. Only 20.9% of clinicians believed that radiation from CT scanning may increase cancer risk in children. About 33.6% of the clinicians reported that they ordered CT scans to investigate suspected head injury due to the poor doctor-patient relationship in China, and to protect themselves against any medical lawsuits in the future. About 80% of the clinicians reported that there are no existing pediatric TBI treatment guidelines in China. Instead a senior doctor’s advice is the most reported guidelines regarding treating pediatric TBI (66.0%). All of the surveyed clinicians reported that the lack of diagnosis and/or treatment standard is the biggest problem in effectively diagnosing and treating pediatric TBI in China. Developing guidelines for the diagnosis and treatment of children with TBI is a high priority in China. The extremely high usage of CT for pediatric TBI in China suggests that it is important to establish evidence-based clinical decision rules to help Chinese clinicians make diagnostic and therapeutic decisions during their practice in order to identify children unlikely to have a clinically-important TBI who can be safely discharged without a CT scan.

## Introduction

Traumatic brain injury (TBI) is the leading cause of death and disability in children around the world, and accounts for approximately half of all trauma deaths [1]. It is estimated that the annual incidence of head injury (HI) for children aged 0–4, 5–9, and 10–14 is 1850/100 000, 1100/100 000 and 1170/100 000, respectively [2]. Each year, over 600,000 children 18 years and younger in the United States and 500,000 to 700,000 children under the age of 15 in England and Wales attend emergency departments (EDs) with a HI [3,4]. Most HI patients recover without specific intervention, yet others have long-term disability or even die from intracranial complications which could potentially have been minimized or avoided by early diagnosis and appropriate treatment.

Mild TBI accounts for about 90% of all TBIs, and is a very common reason for ED presentation [1]. However, the consequences of mild TBI are often not mild [5]. Appropriate clinical guidelines can provide recommendations regarding patient management for clinicians to treat patients with mild TBI. Currently, most of clinical guidelines are for adult TBIs, and there are few guidelines to help clinicians care for children with mild TBI. In 2012, guidelines for the acute medical management of severe traumatic brain injury in infants, children, and adolescents-second edition were published [6]. These international guidelines reflect the current knowledge about pediatric severe TBI, and aim to help improve diagnosis, treatment, and outcomes for children with severe TBI. As such it is critical that clinicians around the world, particular in developing countries, become familiar with these guidelines.

Cranial computed tomography (CT), the reference standard for diagnosis of TBI, is commonly used in the assessment of children with HI in the EDs. However, previous studies reported that less than 10% of patients with suspected TBIs who received CT resulted in a diagnosis of TBI [7,8]. There are substantial variations from country to country regarding the usage of CT in the evaluation of children with HI [9, 10]. According to data from the American Centers for Disease Control and Prevention, about 50% of children assessed in North American EDs for HI underwent CT in 2005, which has more than doubled since the year of 1995[11–13]. CT rates ranged from 6% to 26% across different hospitals in Canada, and increased to 15% to 53% between 1995 and 2005 [14,15]. Studies from UK reported CT proportions of 3.3% to 4.4% at different hospitals [16,17].

Although cranial CT can provide rapid identification and guide management for patients with HI, reduction of CT scans is essential due to the risk of ionizing radiation-induced malignancies [13,18,19]. Previous studies estimated that the rate of lethal malignancies from pediatric cranial CT is between 1 in 1,000 and 1 in 5,000 CT scans, and the risk increases as age decreases [18,19]. It is estimated that 1 in 1,200 children undergoing CT will die due to malignancy from radiation exposure [20]. In addition, CT scans have other disadvantages including the frequent requirement for sedation and increased utility of healthcare resources [21,22].Thus, it should be selectively used to help minimize unnecessary radiation exposure and resource use [21].

Currently, there is no widely accepted protocol for diagnosing children with HI, and little consensus has been reached with regards to appropriate criteria for CT use, especially for mild HI [14, 20]. Findings from previous studies indicated that there is large variability between various protocols and is thus a very common dilemma in the management of HI in children [9,13,14,23]. In order to optimize the balance between identifying clinically important TBI (requiring intervention such as intubation, neurosurgery and hospital admission) and minimizing the cancer risk associated with cranial CT, several evidence-based clinical decision rules (CDRs) for pediatric HI have been developed to help clinicians identify children with mild TBI who can be observed without CT in developed countries [9,13,14–16,21,23–25]. Among those CDRs, the Children's Head Injury Algorithm for the Prediction of Important Clinical Events (CHALICE) from the UK [16], the Canadian Assessment of Tomography for Childhood Head Injury (CATCH) from Canada [15], and the prediction rule for the identification of children at very low risk of clinically important traumatic brain injury developed by the Pediatric Emergency Care Applied Research Network (PECARN) from the U.S. have been identified as being of high quality and accuracy [13,23,25].

In China, reliable data about CT usage in pediatric TBI cases are unavailable. Our recent study conducted at a large children’s hospital showed that 96.9% of children in EDs with mild TBI had a CT, which is much higher than in the USA, Canada and UK [26]. So far, pediatric TBI in China has not received the same attention as adult TBI. Few studies about TBI in Chinese children have been published. Important questions such as how clinicians diagnose and treat pediatric TBI, and what guidelines they follow are not examined in any previous research in China. Since children with HI are often first seen and treated by emergency physicians, in this study we conducted a survey among clinicians at EDs and neurosurgery departments (NDs) in order to gain an understanding of clinician’s knowledge and experiences in diagnosing and treating pediatric TBI, and whether there are clinical guidelines or CDRs that guide their clinical practices.

## Materials and Methods

### Participants and Settings

We conducted a questionnaire survey among clinicians who work at EDs and NDs in 9 large hospitals in China. First, we identified all children’s hospitals and identified where they were distributed geographically (Eastern, Western, Southern, or Northern region in China).We included the largest children’s hospitals in each of the four regions. We then selected one or two academic tertiary hospitals in each of the regions to create a convenience sample. Full-time ED and ND clinicians from the participating sites were invited to fill out the questionnaire survey. The study was approved by the Institutional Review Board of the Capital Medical University School of Public Health, and Wuhan Children’s Hospital. Since clinicians completed the survey questionnaire anonymously, and data were analyzed anonymously, there was no informed consent obtained from the participants.

### Survey Questionnaire

The survey questionnaire consisted of two sections. The first section collected demographic information of the participated ED and ND clinicians, and included questions such as age, gender, professional title, department and years of medical practice in trauma centers. The second section surveyed the responder’s knowledge and experiences in diagnosis and treatment of pediatric TBI. The responder was asked: (1) Is scalp hematoma a type of TBI; (2) Is there an uniform diagnostic criteria for pediatric TBI in China; (3) How do you diagnose TBI in children; (4) Do you know of the pediatric Glasgow Coma Scale(GCS); (5) Which age range is applicable for pediatric GCS; (6) Does your hospital routinely require CT scanning for the diagnosis of pediatric TBI; (7) What best represents the proportion of CT examinations you ordered for children with HI in your practice; (8) Do you know radiation from CT scans could increase cancer risk to children; (9) Is it true that the younger the children are, the stronger the negative impact of CT radiation will be; (10) What is the main reason that you order CT scans to investigate head injury in children; (11) Are there any guidelines for the treatment of pediatric TBI in China; (12) How do you treat pediatric TBI; (13) In your opinion, what is the most important problem regarding pediatric TBI in China. The questionnaire was first piloted among 10 clinicians at Beijing Children’s Hospital and Wuhan Children’s Hospital. The final questionnaire was finalized using feedback from the pilot study.

### Survey Procedure

Two of the researchers (Dr. Fei Di and Huiping Zhu) mailed a packet containing an invitation letter, the survey questionnaires, and a study protocol to a project contact at each of the 9 selected hospitals. The project contact person administered the surveys to all the physicians in his/her hospital. The participants were permitted to complete the survey questionnaire within one week. Finished questionnaire was returned anonymously to the project contact person. The completed questionnaires were then mailed to the research team at the School of Public Health of Capital Medical University for data entry, management, and analysis.

### Data Analysis

Analyses were performed using SAS 9.4 (SAS Institute, Cary, NC). Age statistics were analyzed by mean, minimum, and maximum values. The distributions of responses to other questions were presented by frequency and percentages. Clinical knowledge and experiences of pediatric TBI reported by the surveyed clinicians from four geographical areas in China were compared using chi-square test. Any *P*-value less than or equal to 0.05 was considered statistically significant.

## Results

A total of 235 clinicians completed questionnaires. The age of the surveyed clinicians ranged from 22 to 59 years, with a mean of 34 years (Table 1). About 85.5% of the clinicians were males, and 53.2% clinicians were from NDs. More than 46% of clinicians reported a title of resident doctor, and 28.5% of the clinicians reported more than 10 years of experience in trauma practice.

**Table 1:** Demographic Information of Surveyed ED and ND Clinicians from 9 Large Hospitals in China.

	East		West		South		North		Total	
	n	(%)	n	(%)	n	(%)	n	(%)	n	(%)
**Total**	57		49		66		63		235	
**Age** (mean, range)	35.7	(25, 52)	32.7	(24, 53)	32.0	(22, 51)	35.3	(24, 59)	33.9	(22, 59)
**Gender**										
Male	48	84.2	41	83.7	53	80.3	59	93.7	201	85.5
Female	9	15.8	8	16.3	13	19.7	4	6.3	34	14.5
**Department**										
ED	21	36.8	32	65.3	32	48.5	25	39.7	110	46.8
ND	36	63.2	17	34.7	34	51.5	38	60.3	125	53.2
**Title**										
Resident doctor	21	36.8	25	51.0	38	57.6	26	41.3	110	46.8
Attending doctor	23	40.4	16	32.7	20	30.3	19	30.1	78	33.2
Associate chief physician	12	21.0	6	12.2	6	9.1	11	17.5	35	14.9
Chief physician	1	1.8	2	4.1	2	3.0	7	11.1	12	5.1
**Years of medical practice in trauma**										
<1 year	16	28.1	7	14.3	13	19.7	9	14.3	45	19.1
1–4 years	10	17.5	22	44.9	28	42.4	17	27.0	77	32.8
5–9 years	9	15.8	12	24.5	13	19.7	12	19.0	46	19.6
10+ years	22	38.6	8	16.3	12	18.2	25	39.7	67	28.5

Tables 2 and 3 present clinicians' knowledge and experiences of pediatric TBI stratified by region and title of doctor, respectively. Overall 43.8% of the surveyed clinicians reported a child who only had scalp hematoma without any other signs and symptoms of concussion as a TBI case. Most clinicians (85.1%) reported there were no uniform diagnostic criteria for pediatric TBI in China. In addition, 78.3% of the clinicians claimed that they use adult TBI diagnostic criteria in their clinical practice, and 20.9% clinicians reported they do not know how to diagnose pediatric TBI correctly (data not shown). 148 clinicians (63.0%) reported to know of pediatric GCS, but only 21.6% (32/148) of the clinicians correctly reported the age range applicable for it. The majority of clinicians (91.9%) reported that their hospitals routinely require CT in all patients with suspected head injury. 85.5% of the clinicians reported the proportion of CT scanning they ordered was over 91% for children with a suspected head injury. Only 20.9% of clinicians believed that radiation from CT scanning can increase cancer risk to children, and 36 surveyed clinicians believed that cancer risk increases as age decreases. About 80% of the clinicians reported that there was no treatment guideline existing for pediatric TBI in China. The surveyed clinicians from the four geographic areas did not differ significantly with respect to the knowledge and experiences except for awareness regarding pediatric GCS. Also, there was no significant difference among different levels of doctors in terms of the knowledge and experiences in pediatric TBI.

**Table 2:** Clinicians' Knowledge and Experiences of Pediatric TBI from 9 Large Hospitals in China Stratified by Region.

	East		West		South		North		Total		*P*
	n	(%)	n	(%)	n	(%)	n	(%)	n	(%)	
Total	57		49		66		63		235		
Is scalp hematoma a type of TBI											0.186
Yes	18	31.6	23	46.9	33	50.0	29	46.0	103	43.8	
No	39	68.4	26	53.1	33	50.0	34	54.0	132	56.2	
Is there an uniform diagnostic criteria for pediatric TBI in China											0.723
Yes	9	15.8	7	14.3	12	18.2	7	11.1	35	14.9	
No	48	84.2	42	85.7	54	81.8	56	88.9	200	85.1	
Do you know pediatric GCS											0.015
Yes	31	54.4	27	55.1	40	60.6	50	79.4	148	63.0	
No	26	45.6	22	44.9	26	39.4	13	20.6	87	37.0	
Does your hospital routinely require CT scanning for the diagnosis of pediatric TBI											0.236
Yes	53	93.0	44	89.8	58	87.9	61	96.8	216	91.9	
No	4	7.0	5	10.2	8	12.1	2	3.2	19	8.1	
What best represents the proportion of CT examinations you ordered for children with HI in your practice											0.468
0–60%	3	5.3	2	4.1	4	6.1	1	1.6	10	4.3	
61–80%	3	5.3	2	4.1	2	3.0	1	1.6	8	3.4	
81–90%	2	3.4	4	8.2	2	3.0	8	12.7	16	6.8	
≥91%	49	86.0	41	83.6	58	87.9	53	84.1	201	85.5	
Do you know radiation from CT scans could increase cancer risk to children											0.806
Yes	13	22.8	11	22.4	11	16.7	14	22.2	186	79.1	
No	44	77.2	38	77.6	55	83.3	49	77.8	49	20.9	
Are there any guidelines for the treatment of pediatric TBI in China											0.603
Yes	9	15.8	10	20.4	17	25.8	13	20.6	49	20.9	
No	48	84.2	39	79.6	49	74.2	50	79.4	186	79.1	

**Table 3:** Clinicians' Knowledge and Experiences of Pediatric TBI from 9 Large Hospitals in China Stratified by Doctor’s Title.

	Resident doctor		Attending doctor		Associate chief physician		Chief physician		Total		*P*
	n	(%)	n	(%)	n	(%)	n	(%)	n	(%)	
Total	110		78		35		12		235		
Is scalp hematoma a type of TBI											0.299
Yes	54	49.1	33	42.3	13	37.1	3	25.0	103	43.8	
No	56	50.9	45	57.7	22	62.9	9	75.0	132	56.2	
Is there an uniform diagnostic criteria for pediatric TBI in China											0.576
Yes	14	12.7	15	19.2	5	14.3	1	8.3	35	14.9	
No	96	87.3	63	80.8	30	85.7	11	91.7	200	85.1	
Do you know pediatric GCS											0.615
Yes	69	62.7	46	59.0	24	68.6	9	75.0	148	63.0	
No	41	37.3	32	41.0	11	31.4	3	25.0	87	37.0	
Does your hospital routinely require CT scanning for the diagnosis of pediatric TBI											0.237
Yes	105	95.5	69	88.5	32	91.4	10	83.3	216	91.9	
No	5	4.5	9	11.5	3	8.6	2	16.7	19	8.1	
What best represents the proportion of CT examinations you ordered for children with HI in your practice											0.836
0–90%	18	16.4	10	12.8	5	14.3	1	8.3	34	14.5	
≥91%	92	83.6	68	87.2	30	85.7	11	91.7	201	85.5	
Do you know radiation from CT scans could increase cancer risk to children											0.196
Yes	86	78.2	67	85.9	24	68.6	9	75.0	186	79.1	
No	24	21.8	11	14.1	11	31.4	3	25.0	49	20.9	
Are there any guidelines for the treatment of pediatric TBI in China											0.138
Yes	21	19.1	16	20.5	6	17.1	6	50.0	49	20.9	
No	89	80.9	62	79.5	29	82.9	6	50.0	186	79.1	

About 33.6% of the clinicians reported that they ordered CT scans to investigate head injury in children due to poor doctor-patient relationship and self-protection in their practice (Table 4). A senior doctor’s guidance was the most reported basis used to treat pediatric TBI (66.0%) (Table 5). Finally, with regards to what is the most important problem in managing pediatric TBI in China, all of the surveyed clinicians claimed the lack of diagnosis and/or treatment guidelines is the biggest problem.

**Table 4:** The Main Reason for Ordering CT Scans to Investigate Head Injury for Children Reported by Surveyed Clinicians in China.

Reason	n	(%)
The tense doctor-patient relationship and self-protection considerations	79	33.6
Patients showed indications for having a CT scan^a^	156	66.4

^a^ Indications include headache, vomiting, seizure, intoxication, short-term memory deficit, suspected skull fracture, serious trauma mechanism, etc.

**Table 5:** Routine Basis for Treatment of Pediatric TBI Reported by Surveyed Clinicians in China.

Basis for treatment of pediatric TBI^a^	n	(%)
Own experience	80	34.0
Senior doctor's guidance	155	66.0
Clinical practice guidelines developed in each hospital	114	48.5
Clinical practice guidelines for adults' TBI developed by The Ministry of Health of the People's Republic of China	51	21.7

^*a*^ This is a multiple choice question, so the total percentage is more than 100%.

## Discussion

Results of our study showed that 43.8% of the surveyed clinicians reported children with only scalp hematoma as TBI cases, and 20.9% of the clinicians reported they do not know how to diagnose pediatric TBI correctly. This study’s findings indicate that knowledge regarding pediatric TBI among Chinese clinicians is limited. The U.S. Centers for Disease Control and Prevention defines TBI as an occurrence of injury to the head with decreased level of consciousness, amnesia, and/or neurologic or neuropsychological dysfunction or diagnosis of intracranial lesion [27]. The occurrence of scalp hematoma without a concussion should not be diagnosed as a TBI [28]. Chinese clinicians need to be aware of the diagnostic definitions in order to properly diagnose pediatric TBI for proper treatment.

Data from North American research studies showed significant variation in CT scan ordering for pediatric head injury in North American hospitals [11–14]. Yet, there is considerable homogeneity in the significant high utilization of CT scan in China. Our results demonstrated that an overwhelming majority of EDs and NDs clinicians (85.5%) reported the proportion of CT examinations they ordered for children with suspected head injury was over 91%. This rate did not differ across the participating hospitals from the four geographic regions. In our study, 91.9% of the clinicians reported that performing CT in all children with suspected head injury is required in their hospitals, suggesting that the procedure of performing CT in all HI patients is universal in China. This high frequency in ordering CT scans by clinicians most likely relates to the ease of obtaining head CT scans in hospitals in China. Deteriorating medical environment further contributes to the increased usage of CT scans, as reported by the surveyed clinicians. The Lancet recently published several editorials about the deteriorated Chinese medical situation and the complex and poor doctor-patient relationship. These editorial comments pointed out that personal safety is of greater concern for many Chinese doctors now because they are often victims of terrible violence [29,30]. Our study showed that 33.6% of the surveyed clinicians reported that they order CT scans indiscriminately in their practice due to the tense doctor-patient relationship and self-protection considerations. Most of the surveyed clinicians reported that they cannot afford the consequences if a child with suspected head injury is misdiagnosed without an undergoing CT scan.

There is heightened concern about radiation exposure as patients with head injury tend to be young, and they may experience multiple head injuries that require repeated CT scans during their lifetime, thus giving them higher radiation risks [19]. Brody et al. found that children are at higher risk of cancer and central nervous system damage from radiation from CT scans than adults [31]. Hessetal. also reported CT scans expose children to ionizing radiation, which has been linked with the development of brain tumors, leukemia and other cancers [32]. In addition, the cancer risk from exposure to radiation is different between children and adults for a given dose of radiation because children are much more sensitive to radiation effects [31,22,33]. The current consensus among clinicians is that the increased lifetime risk of cancer from CT scans is greater among the younger child than adults [13,19,33]. In our study, we found that only 20.9% of clinicians believed that radiation from CT scans could increase cancer risk to children, and 36 surveyed clinicians reported the cancer risk increases as age decreases. Previous studies concluded that the possible risk of cancer from childhood CT scans is not clearly understood by health care professionals [31,34,35]. Lee et al. surveyed ED physicians and found only 9% of physicians believed that the lifetime risk of cancer can be increased from CT scans [34]. Jacobetal. Also found that only 12.5% of surveyed physicians in the United Kingdom were aware of the potential association between CT radiation and cancer [35].

Over the past decade, the number of CT scans for head injury in children has increased in many countries [11,12,14]. The increased CT uses not only expose a large number of children to the risk of radiation but also add substantially to health care costs each year, especially in children with very low risk of TBI [15]. Furthermore, the diagnostic yield has remained low, since many TBIs identified with CT do not need acute intervention, and some are non-traumatic findings or even false positives [13]. Previous studies showed that only 4%-7% children with mild head injury have a visible brain damage on CT, and only 0.5% have an intracranial lesion requiring acute neurosurgical intervention [14,36]. The appropriate way of reducing missed clinically important intracranial injuries and unnecessary CT scan is to use CDRs. Smits et al. reported that selecting patients with mild head injury for CT scan on the basis of CDRs was the most cost-effective procedure, resulting in annual U.S. cost savings of $120 million when comparing with scanning all patients with mild head injury [37]. Therefore, selective CT scan based on CDRs is desirable for children with head injury, especially in the context of a busy and overcrowded healthcare delivery system in China.

In our study, 85.1% of the clinicians reported there were no uniform diagnostic criteria for children with TBI in China, and 78.3% of them claimed they use adult diagnostic criteria in their clinical practice. However, patterns of injury in children are often different from those in adults. For example, children are more likely to sustain calvarial fractures due to a larger craniofacial ratio and thinner skull [22]. Also, mechanisms of abused TBI in children do not typically occur in adults [22]. Furthermore, the severity of TBI is assessed by Glasgow Coma Scale (GCS). Yet, many assessments for an adult patient would not be appropriate for infants and young children, as the standard GCS has limited applicability for children below the age of 5 years, and thus has been modified slightly to create the pediatric GCS [38]. Our results showed that 148 (63%) surveyed clinicians reported they know the pediatric GCS, but only 21.6% reported the age range applicable for it correctly.

About 80% of the surveyed clinicians reported neither treatment guidelines nor CDRs about pediatric TBI exist in China, and all of the clinicians claimed the biggest problem in treating pediatric TBI is the lack of diagnosis and treatment guidelines. They recognized the first priority is to develop guidelines for the diagnosis and treatment of children with TBI. Guidelines can help clinicians cope with the uncertainty of medical decision making. A study from American CDC found that routine use of guidelines for the treatment of TBI patients could substantially reduce the deaths and medical, rehabilitative, and societal costs [39]. Although there are some guidelines and CDRs for pediatric TBI, they were developed outside of China and most of clinicians in China are not aware of them or could not read them due to language barriers. Thus, it would be ideal that clinicians in China develop clinical guidelines or CDRs that fit the specific needs of local healthcare environments to help improve the care of children with TBI. In addition, the extremely high rate of ordering CT in China suggests that it is important to establish CDRs similar to CATCH, CHALICE, or PECARN to help guide diagnostic and therapeutic decision of Chinese clinicians so that it is possible to better identify clinically-important TBI that can be safely discharged without a CT scan.

Our study has some limitations. First, clinicians in the EDs and NDs were selected from 9 major hospitals in China, which was just a convenience sample. We did not use random sampling, so it is possible that this could have selection bias, and would be not representative of all EDs and NDs clinicians in China. Nevertheless, the including hospitals in our study are the largest children’s hospitals in eastern, western, southern and northern regions of China, or one or two academic tertiary hospitals in each of the areas, thus the participating doctors would have the most advanced knowledge and clinical experiences. We believe that the survey results reflect the common practices in China as we found little variation in their knowledge and practices across regions. Second, we did not specifically analyze the doctors’ education background, since the overwhelming majority of the doctors in the 9 hospitals have very similar education background.
